# Reliability of Vibroarthrography to Assess Knee Joint Sounds in Motion

**DOI:** 10.3390/s20071998

**Published:** 2020-04-02

**Authors:** Kristin Kalo, Daniel Niederer, Rainer Sus, Keywan Sohrabi, Volker Groß, Lutz Vogt

**Affiliations:** 1 Department of Sports Medicine and Exercise Physiology; Goethe University Frankfurt am Main, 60487 Frankfurt am Main, Germany; niederer@sport.uni-frankfurt.de (D.N.); l.vogt@sport.uni-frankfurt.de (L.V.); 2 Faculty of Health Sciences, University of Applied Sciences, 35390 Giessen, Germany; rainer.sus@ges.thm.de (R.S.); keywan.sohrabi@ges.thm.de (K.S.); volker.gross@ges.thm.de (V.G.)

**Keywords:** acoustic emission, measurement properties, vibroarthrographic, knee sound, knee noise, crepitation, crepitus

## Abstract

Knee acoustic emissions provide information about joint health and loading in motion. As the reproducibility of knee acoustic emissions by vibroarthrography is yet unknown, we evaluated the intrasession and interday reliability of knee joint sounds. In 19 volunteers (25.6 ± 2.0 years, 11 female), knee joint sounds were recorded by two acoustic sensors (16,000 Hz; medial tibial plateau, patella). All participants performed four sets standing up/sitting down (five repetitions each). For measuring intrasession reliability, we used a washout phase of 30 min between the first three sets, and for interday reliability we used a washout phase of one week between sets 3 and 4. The mean amplitude (dB) and median power frequency (Hz, MPF) were analyzed for each set. Intraclass correlation coefficients (ICCs (2,1)), standard errors of measurement (SEMs), and coefficients of variability (CVs) were calculated. The intrasession ICCs ranged from 0.85 to 0.95 (tibia) and from 0.73 to 0.87 (patella). The corresponding SEMs for the amplitude were ≤1.44 dB (tibia) and ≤2.38 dB (patella); for the MPF, SEMs were ≤13.78 Hz (tibia) and ≤14.47 Hz (patella). The intrasession CVs were ≤0.06 (tibia) and ≤0.07 (patella) (*p* < 0.05). The interday ICCs ranged from 0.24 to 0.33 (tibia) and from 0 to 0.82 (patella) for both the MPF and amplitude. The interday SEMs were ≤4.39 dB (tibia) and ≤6.85 dB (patella) for the amplitude and ≤35.39 Hz (tibia) and ≤15.64 Hz (patella) for the MPF. The CVs were ≤0.14 (tibia) and ≤0.08 (patella). Knee joint sounds were highly repeatable within a single session but yielded inconsistent results for the interday reliability.

## 1. Introduction

Acoustic emissions are seen as an indicator for joint conditions [1]. Unphysiological changes in cartilage, for example, are reported to lead to characteristical alterations in the joint sound during active movement [2]. McCoy et al. [3] referred to the recording of vibrations caused by joint articulation through local accelerometers on the skin as vibroarthrography.

A selective classification of osteoarthritis stages via vibroarthrography seems possible [4,5]. Some studies using vibroarthrography claim an even better detection of knee osteoarthritis stages than with the current imaging techniques [2,5]. Results by Befrui et al. [5] revealed a specificity of 80% and a sensitivity of 75% for the early diagnosis of osteoarthritis in comparison to symptom-free knee joint sounds. Kiselev et al. [2] showed a diagnostic accuracy of the acoustic emissions measurement from good to very good. Moreover, knee sounds produced by the articulation of joint components not only contain information on the joint health and morphology, but also have been well discussed as surrogates of knee joint loading during motion [1,4,6,7,8]. These findings appear to be particularly important, because vibroarthrography presents a new perspective on knee arthrokinematics. Aside from providing information on structural joint health, like traditional diagnostic techniques, knee joint sounds may additionally indicate functional changes in knee joint cartilage [9,10].

To thoroughly assess the progression of knee osteoarthritis or the influence of different acute or long-term therapies on knee joint health, a reproducible tool is needed. In a first study on this topic, Teague et al. [11] investigated the consistency of main acoustic events during repetitive motions which occurred at consistent joint angles. This work provided the initial step in examining the quality of acoustic emission measurements but was not sufficient to assess measurement reliability.

No other study investigated the reproducibility of vibroarthrographic knee joint assessments. Therefore, we determined the intrasession and interday reliability of knee joint vibroarthrography in motion. 

## 2. Methods

### 2.1. Study Design and Ethics

The present study followed the Guidelines for Reporting Reliability and Agreement Studies (GRRAS) [12]. It was conducted in accordance with the Declaration of Helsinki (with its recent modification at Fortaleza, 2013), and approval of the local ethics committee was obtained. Each participant signed an informed consent prior to study inclusion.

### 2.2. Participants

Nineteen healthy adults (25.6 ± 2.0 years, 11 females, 8 males, body mass index 21.3 ± 2.2 kg/m^2^, 326 ± 181 min of physical activity/sport per week) were recruited by means of personal addressing. Exclusion criteria encompassed a history of knee osteoarthritis, knee injuries in the past year, current knee pain/muscle soreness, other diseases that affected their walking ability/standing stability, and intense physical activity in the last 48 h.

### 2.3. Measurement Protocol

After the study enrollment period, each participant was scheduled for two visits (sessions) with a wash-out phase (time period between measurements) of seven days (no variance), at the same time of day. On day A, the participants performed the measurement three times (three sets) with 30 min between each set, and on day B they performed only one set. Each movement set consisted of five repetitions of standing up and sitting down, surrounded by two calibration phases. During the calibration phases, the participants rested for 5 s in the starting/ending position. For the five repetitions, the participants first moved from a seated position on a height-adjustable bench with a 90° knee, ankle, and hip angle, to an upright standing position (knee angle of 0°), then back to the sitting position (knee angle of 90°). The movement speed/frequency was standardized using a visual metronome with the following trigger: 2 s for standing up and 2 s for sitting down, in a flowing movement. The leg (side) to be assessed during the measurements was randomly selected and identical for every session.

### 2.4. Sound Signal Assessment, Extraction, and Processing

Knee joint sounds/acoustic emissions were recorded using two high-performance, low-power, and top-port silicon acoustic sensors (microphone size: 3 cm in diameter and 1 cm in depth, SPU0414HR5H-SB, Knowles Electronics, LLC., Itasca, IL, USA); one placed on the medial tibial plateau (upper limit of the sensor 2 cm below the joint space) and one in the center of the patella. Both locations were palpated, equidistant to reach centering, and controlled by a second investigator. These specific anatomical locations offer an optimal contact area, which is the closest to the bone surface, minimizing the influence of skin and subchondral soft tissue on the generated acoustic signal [5,13]. The microphones were placed in a standing position and attached to the skin using double-sided tape (4 cm, LSM2-ZKP-023, Löwenstein Medical Diagnostics GmbH, Germany). Beforehand, the area of skin to be tested was prepared (if necessary) by hair removal and cleaned with alcohol. The sound signals were digitized with a sampling rate of 16,000 Hz and transferred to a computer through an A/D converter (USB-6009, 8 AI (14-bit, 48 kS/s), 2 AO (150 Hz), 13 DIO USB Multifunction I/ODevice, National Instruments Corp., Austin, TX, USA). The A/D converter was placed in a belt bag around the participant’s waist, and wires were attached to the thigh to allow for natural movement. 

**Figure 1:**
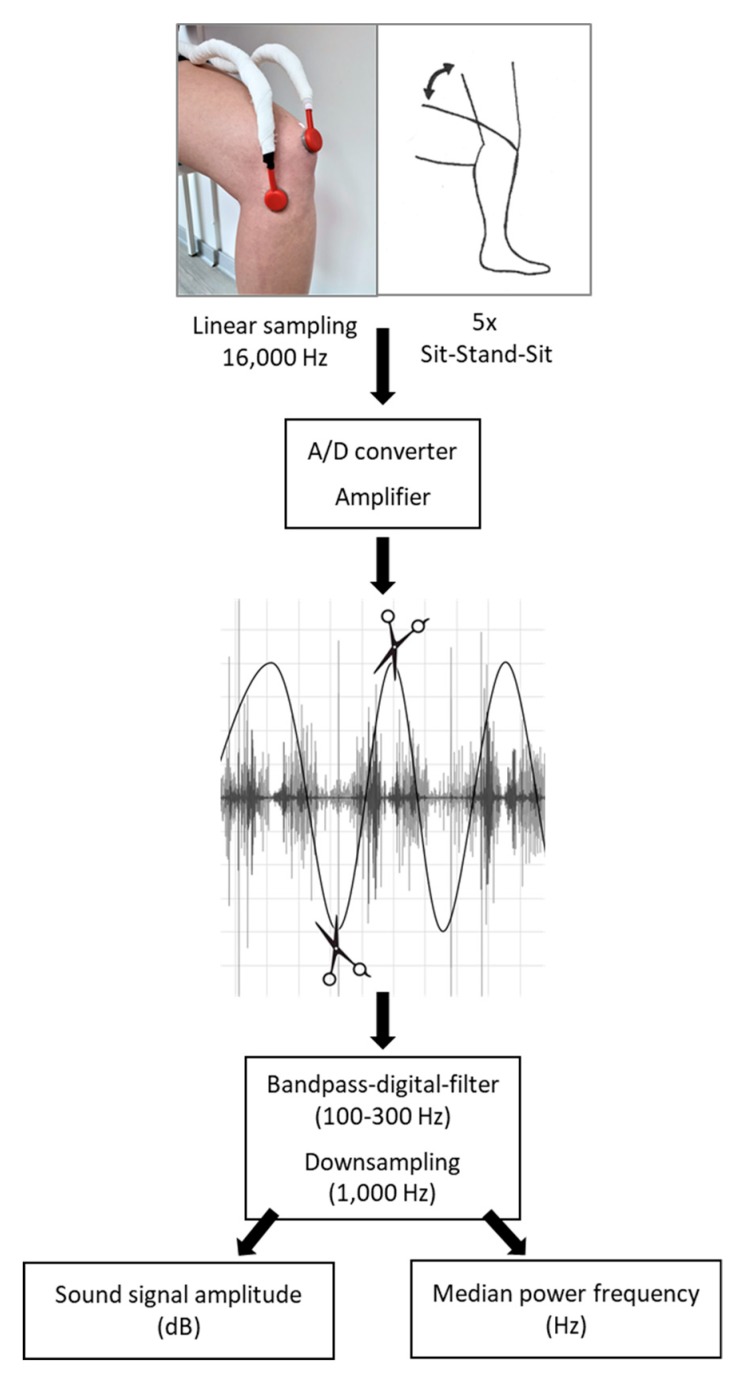
Sound data extraction and processing. A set consisted of five movement cycles (standing up and sitting down); data from the same movements were averaged. A/D: analog digital converter.

We extracted the single standing up/sitting down movements (cycles) to calculate a mean out of the five repetitions. For data extraction and processing, we used Matlab version R2018b (MathWorks, Natick, MA, USA). We filtered the sound signal using a default bandpass digital filter of Matlab (version R2018b) with cut-off frequencies of (lower) 100 Hz and (upper) 300 Hz. The knee sound recording and data processing (including filtering and downsampling) are depicted in Figure 1.

### 2.5. Statistics

All statistical calculations were made with SPSS version 25 (SPSS Inc., Chicago, USA) and Microsoft Excel 2013 for Windows (Microsoft Corporation, Redmond, USA). In our study, the term intrasession is defined as repeated measurements during one day without replacing the microphones. However, the term interday describes the same measurements on two different days with replacement of the microphones. To determine intrasession and interday reliability, intraclass correlation coefficients (ICCs (2,1)) with 95% confidence intervals were calculated for all measurements of each session. The calculations for the intrasession reliability contained all three sets at day A; for the interday reliability calculation, the first set of day A was compared with the only set of day B. Resulting ICC values were interpreted according to Fleiss [14] as “poor” (<0.40), “fair to good” (0.40–0.75), and “excellent” (>0.75). For the relative test–retest reliability, the standard error of measurement (SEM) was estimated using the formula “SEM = standard deviation × √(1 – ICC)” [15]. The SEM of the median power frequency (MPF) was also expressed as a percentage of the corresponding mean sound signal value (SEM%). The typical errors of intrasession and interday measurements were analyzed using the coefficient of variation (CV = standard deviation/mean). In accordance with Albertus-Kajee et al. [16], CV values less than 12% were defined as “acceptable”. In all analyses, the level of significance was set at *p* < 0.05.

## 3. Results

No participant had to be excluded for the intrasession reliability analysis. Two participants had to be excluded for the interday reliability analysis because they had displayed an exclusion criterion (delayed onset muscle soreness) on the second measurement day. No one withdrew informed consent.

### 3.1. Acoustic Emission Values

**Figure 2:**
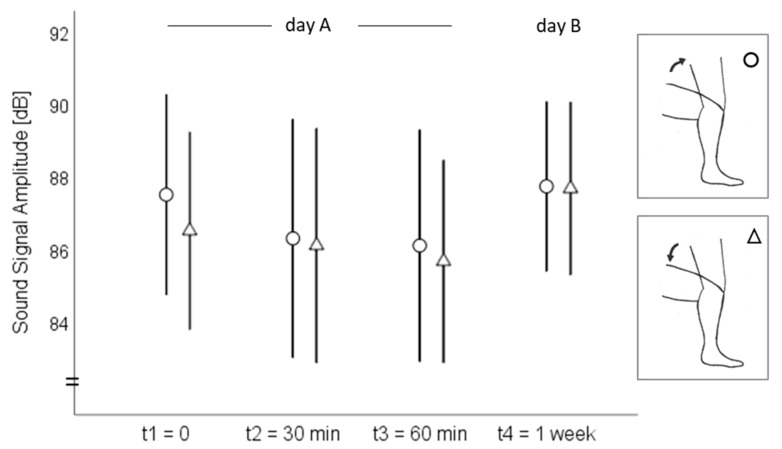
Means and 95% confidence intervals of the sound signal amplitude (dB) at the medial tibial plateau during standing up (circles) and sitting down (triangles).

**Figure 3:**
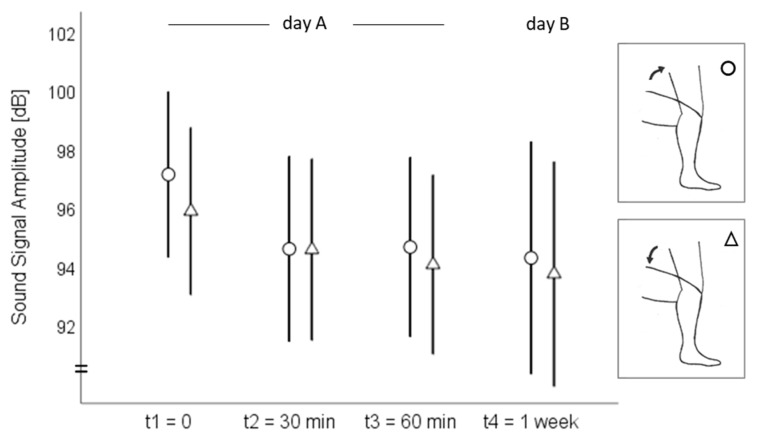
Means and 95% confidence intervals of the sound signal amplitude (dB) at the patella during standing up (circles) and sitting down (triangles).

**Figure 4:**
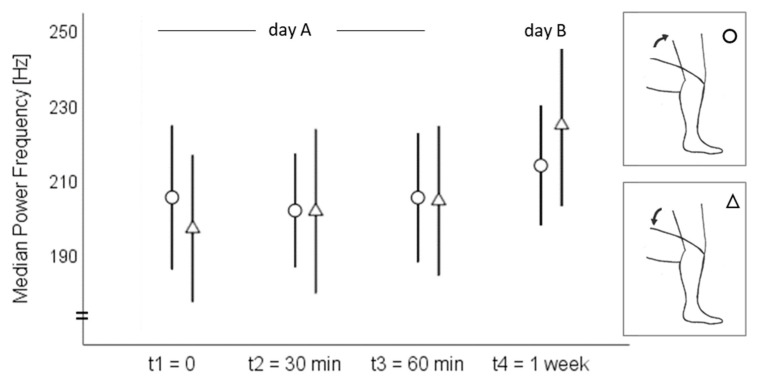
Means and 95% confidence intervals of the median power frequency (Hz) at the medial tibial plateau during standing up (circles) and sitting down (triangles).

**Figure 5:**
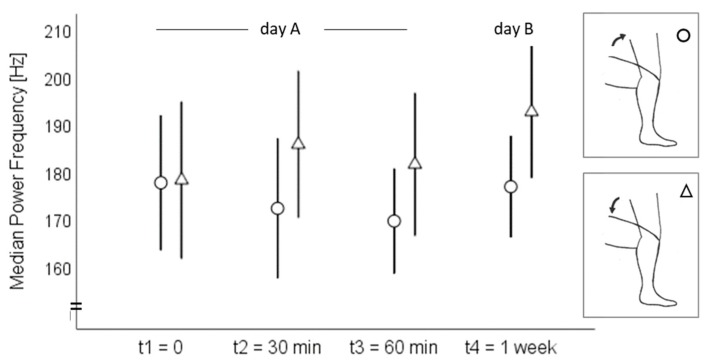
Means and 95% confidence intervals of the median power frequency (Hz) at the patella during standing up (circles) and sitting down (triangles).

On day A, the sound signal amplitude ranged from 68 to 99 dB at the medial tibial plateau and from 76 to 104 dB at the mid-patella during both the standing up and sitting down movements (Figure 2). The MPF ranged from 106 to 298 Hz at the medial tibia and from 113 to 244 Hz at the patella (Figure 3). On day B, the amplitude ranged from 80 to 96 dB at the medial tibia (Figure 2) and from 71 to 103 dB at the patella (Figure 3) during the standing up and sitting down movements. The MPF ranged from 152 to 323 Hz at the medial tibia (Figure 4) and from 142 to 231 Hz at the patella (Figure 5) during the whole standing up and sitting down task.

### 3.2. Intrasession Reliability

The ICCs ranged from 0.85 to 0.95 at the medial tibial plateau and from 0.73 to 0.87 at the patella (*p* < 0.05) for both MPF and amplitude during standing up and sitting down. The corresponding values, as well as SEMs and CVs for the intrasession reliability, are displayed in Table 1.

### 3.3. Interday Reliability

The ICCs ranged from 0.24 to 0.33 at the medial tibial plateau and from 0 to 0.82 at the patella for both MPF and amplitude during standing up and sitting down. The corresponding values, as well as SEMs and CVs for the interday reliability, are displayed in Table 2.

## 4. Discussion

### 4.1. Intrasession Reliability

We found excellent values for intrasession reliability of the amplitude and the MPF at both sensor positions. As there are no comparable studies for reliability in joint sound recordings available, other types of assessments are needed for discussing our reliability indices. We thus considered reliability indices of other superficial assessments as relevant; for example, EMG electrodes have a similar adhesive behavior, as well as commonly similar outcome parameters such as acoustic emission measurements (MPF and signal amplitude). Most EMG studies likewise revealed excellent intrasession reliability, with indices (ICC) greater than 0.80 [17,18,19,20]. Regarding dynamic contractions, EMG studies proved excellent reproducibility for EMG amplitude and MPF (ICC = 0.83-0.98) [20,21,22]. The ICC values provide a first indication that vibroarthrographic assessments, without removing and replacing the microphones, are reproducible. The SEMs found are in line with the EMG results, which displayed a small SEM for the intrasession amplitude as well as for the MPF [18,23]. Likewise, the intrasession CVs of the amplitude and MPF were acceptable at both sensor positions and are comparable with results found in the EMG research [20].

When applied to previously published results, the intrasession SEM of the amplitude and the MPF were smaller than the mean differences between the sit-to-stand acoustics and other standardized loading conditions, such as walking downstairs (sit-to-stand was quieter) or passive knee flexion–extension (sit-to-stand was louder) [7]. Moreover, mean differences between healthy and osteoarthritic knee sounds in other studies were larger than the SEMs found in the present study [13,24,25]. As the values are larger than the measurement error, the differences between the conditions, as well as between healthy individuals and patients, are thus considered systematic.

### 4.2. Interday Reliability

Overall, the interday reliability displayed inconsistent values depending on the sensor location and outcome parameter. The ICC results suggested poor reliability for all outcome parameters at the medial tibial plateau and for the amplitude at the patella; only the MPF at the patella showed a good-to-excellent interday reliability. With regard to the interday reliability for dynamic movements, EMG literature is also quite contrasting [18]. Mathur et al. [26] showed fair-to-excellent reliability for the MPF (ICC = 0.59 − 0.88) and for the amplitude (ICC = 0.58 − 0.99). Karamanidis et al. [27] analyzed EMG reliability at different step frequencies and found a wide range of reliability values for EMG amplitude (ICC = 0.44 − 0.94) as well as for MPF (ICC = 0.21 − 0.92), which was likewise dependent on sensor location and outcome parameter.

Interday SEM was fair regarding the amplitude and MPF during different loading conditions [4,7]. When comparing healthy and osteoarthritic knees, the interday SEM revealed a good reliability for both outcome parameters [5,25]. By removing and replacing electrodes, Hashemi Oskouei et al. [18] produced a poor ICC (ICC < 0.50), but this did not substantially affect the SEM. This is in line with our results regarding the differences between healthy and osteoarthritic knee joints, which imply that within-subject variance was not affected by electrode removal and replacement between measurement days. The interday CV was also acceptable for the amplitude at both microphones and the MPF at the patella but was low for the MPF at the medial tibial plateau. This is in contrast to EMG studies examining interday reliability, which often reported reduced ICC with increased CV [17,18]. 

### 4.3. Practical Relevance

Intrasession measurements, i.e., without microphone replacement, are well qualified for practical application like cross-sectional or acute-effect (therapy/exercise/training) assessments. In contrast, replacement of microphones leads to poor comparability between measurements. If, in practice or science, a replacement is needed, a certain standardization is necessary. This standardization can be done internally or externally. Internal standardization can be reached by means of a reference measurement, for example, by a zero-load (passive movement) measurement/condition. A possible external standardization would be a baseline measurement, where pre–post changes are considered as dependent variables. 

### 4.4. Limitations and Future Study

Considering the large variance in interday reliability, it is of great relevance to find procedures to increase (and homogenize) the reliability of vibroarthrographic assessment with microphone replacements in between the repeated measures. Future study is warranted to reveal if the intraindividual vibroarthrographic signal can be influenced by, for example, therapeutic applications. More precisely: can the hypothetical benefit of therapies be measured by knee joint acoustic emissions?

## 5. Conclusion

Knee joint sound signals emit an excellent repeatability within a single day and session; in particular, measurements made without replacing the microphones are found to be reproducible. Acute therapy effects or cartilage function, for example, may thus be reliably quantifiable. In contrast, we found inconsistent results for the interday reliability. Skin preparation and the replacement of electrodes may have major influences on the interday repeatability. Consequently, knee joint sounds for investigating the progression of knee osteoarthritis, or the influence of long-term therapies on knee joint health, may be assessed using MPF at the patella. The assessment of other parameters under circumstances where microphone replacement is necessary may only be useful when a reference value can be assessed or when large effects/changes are anticipated.

## Figures and Tables

**Table 1:** Intrasession reliability at the medial tibial plateau and the patella for the standing up and sitting down movement ^1^.

		ICC	95% Confidence Interval		SEM		CV
			Lower Limit	Upper Limit		SEM%	
**Medial tibial plateau**							
**Standing up**	**MPF**	0.85 *	0.71	0.93	13.78 Hz	7	0.06
	**amplitude**	0.95 *	0.89	0.98	1.44 dB		0.02
**Sitting down**	**MPF**	0.93 *	0.85	0.97	11.44 Hz	6	0.05
	**amplitude**	0.94 *	0.88	0.98	1.44 dB		0.02
**Patella**							
**Standing up**	**MPF**	0.73 *	0.51	0.87	14.47 Hz	8	0.07
	**amplitude**	0.86 *	0.72	0.94	2.38 dB		0.02
**Sitting down**	**MPF**	0.87 *	0.74	0.94	11.73 Hz	6	0.06
	**amplitude**	0.85 *	0.72	0.94	2.36 dB		0.02

^1.^ MPF = median power frequency; ICC = intraclass correlation coefficient; amplitude = sound signal amplitude; SEM = standard error of measurement; CV = coefficient of variation; * significance level *p* < 0.05.

**Table 2:** Interday reliability at the medial tibial plateau and the patella for the standing up and sitting down movements ^1^.

		ICC	95% Confidence Interval		SEM	SEM%	CV
			Lower Limit	Upper Limit			
**Medial tibial plateau**							
**Standing up**	**MPF**	0.24	−0.26	0.64	31.27 Hz	15	0.11
	**amplitude**	0.33	−0.16	0.69	4.19 dB		0.04
**Sitting down**	**MPF**	0.33	−0.16	0.69	35.39 Hz	17	0.14
	**amplitude**	0.27	−0.22	0.66	4.39 dB		0.04
**Patella**							
**Standing up**	**MPF**	0.62 *	0.22	0.85	15.64 Hz	9	0.07
	**amplitude**	0.16	−0.33	0.59	6.27 dB		0.05
**Sitting down**	**MPF**	0.82 *	0.56	0.93	13.56 Hz	7	0.08
	**amplitude**	0.00	−0.51	0.42	6.85 dB		0.06

^1.^ MPF = median power frequency; ICC = intraclass correlation coefficient; amplitude = sound signal amplitude; SEM = standard error of measurement; CV = coefficient of variation; * significance level *p* < 0.05.
